# A virulence activator of a surface attachment protein in *Burkholderia pseudomallei* acts as a global regulator of other membrane-associated virulence factors

**DOI:** 10.3389/fmicb.2022.1063287

**Published:** 2023-01-16

**Authors:** Zhenxin Sun, Yun Heacock-Kang, Ian A. McMillan, Darlene Cabanas, Jan Zarzycki-Siek, Tung T. Hoang

**Affiliations:** School of Life Sciences, University of Hawai‘i at Mānoa, Honolulu, HI, United States

**Keywords:** *Burkholderia pseudomallei*, transposon mutagenesis, Tn-Seq, RNA-Seq, bacterial cell envelope, gene regulation, virulence, single-cell transcriptomic

## Abstract

*Burkholderia pseudomallei (Bp)*, causing a highly fatal disease called melioidosis, is a facultative intracellular pathogen that attaches and invades a variety of cell types. We previously identified BP1026B_I0091 as a surface attachment protein (Sap1) and an essential virulence factor, contributing to *Bp* pathogenesis *in vitro* and *in vivo*. The expression of *sap1* is regulated at different stages of *Bp* intracellular lifecycle by unidentified regulator(s). Here, we identified SapR (BP1026B_II1046) as a transcriptional regulator that activates *sap1*, using a high-throughput transposon mutagenesis screen in combination with Tn-Seq. Consistent with phenotypes of the Δ*sap1* mutant, the Δ*sapR* activator mutant exhibited a significant reduction in *Bp* attachment to the host cell, leading to subsequent decreased intracellular replication. RNA-Seq analysis further revealed that SapR regulates *sap1*. The regulation of *sap1* by SapR was confirmed quantitatively by qRT-PCR, which also validated the RNA-Seq data. SapR globally regulates genes associated with the bacterial membrane in response to diverse environments, and some of the genes regulated by SapR are virulence factors that are required for *Bp* intracellular infection (e.g., type III and type VI secretion systems). This study has identified the complex SapR regulatory network and its importance as an activator of an essential Sap1 attachment factor.

## Introduction

*Burkholderia pseudomallei* (*Bp*) is a soil dwelling Gram-negative bacterium that causes the disease melioidosis with considerable morbidity and mortality in a broad range of hosts, including humans, cattle, horses, dolphins, goats, and koalas (Wiersinga et al., 2006; Currie, 2008). This pathogen demonstrates remarkable capabilities of surviving under hostile environments, particularly during interaction with the host immune system (Wiersinga et al., 2012). *Bp* infections can be acquired through three major routes: percutaneous inoculation *via* breaks in skin, inhalation of aerosolized water or soil, and ingestion of contaminated water (Dance, 2000; Wiersinga et al., 2006). *Bp* can disseminate to distant sites resulting in many possible manifestations associated with infection (Wiersinga et al., 2006). The most severe clinical manifestation is melioidosis septic shock where the eradication of *Bp* is difficult (Wiersinga et al., 2006). *Bp* has recently gained much interest from western countries despite having a high prevalence in Southeast Asia and Northern Australia (Inglis et al., 2006; Currie et al., 2008; Limmathurotsakul et al., 2016). *Bp* has been classified as a Tier 1 select agent by the United States. Centers for Disease Control and Prevention (CDC) due to its potential to pose a severe threat to public health and safety (Dance and Alibek, 2005; Gilad et al., 2007).

*Burkholderia pseudomallei* harbors two circular chromosomes totaling 7.25 mega bases, which contain a large number of genes encoding a wide array of virulence factors (Holden et al., 2004; Wiersinga et al., 2006) to promote successful infection of various hosts and survival within phagocytic and nonphagocytic cells (Jones et al., 1996; Harley et al., 1998). Amongst these virulence factors, adhesins (Balder et al., 2010) and surface attachment factors (BPSL0097/BP1026B_I0091 and BPSS1860/BP1026B_II1992; Heacock-Kang et al., 2021), the Bsa type III secretion system cluster 3 (T3SS3; Sun et al., 2010), a modulator of host cell tubulin (BPSS1818/BP1026B_II1948; Heacock-Kang et al., 2021), host cell autophagy evasion factor (BPSS0015/BP1026B_II0016; Heacock-Kang et al., 2021), the type VI secretion system cluster 5 (T6SS-5; Shalom et al., 2007; Burtnick et al., 2011; Chen et al., 2011), the cytotoxin *Burkholderia* lethal factor 1 (Cruz-Migoni et al., 2011), and capsular polysaccharide I (Reckseidler et al., 2001) facilitate *Bp* intracellular infection in the host. Many of these virulence factors are associated with the bacterial membrane. *Bp* initiates its intracellular life cycle by attaching and entering host cells, followed by rapid escape from endocytic vesicles through T3SS3 expression (Warawa and Woods, 2005; Burtnick et al., 2008). Once in the cytosol, *Bp* moves freely using BimA-facilitated polymerization of host cell actin (French et al., 2011; Benanti et al., 2015). Upon contact with host cell membranes, *Bp* induces T6SS-5 mediated membrane fusion with adjacent cells, resulting in multinucleated giant cell (MNGC) formation (Schwarz et al., 2014; Toesca et al., 2014). As recently demonstrated, *Bp* undergoes dynamic gene expression changes while transiting through host cells (Heacock-Kang et al., 2021). Up to 1,953 genes showed differential gene expression patterns in a stage specific manner during *Bp* intracellular infection indicating complex regulatory functions (Heacock-Kang et al., 2021; McMillan et al., 2021). The *Bp* genome is annotated to encode a wide spectrum of regulatory proteins, such as two-component sensor-regulator systems, alternative sigma factors, and different classes of regulatory proteins (Holden et al., 2004). Many of the virulence factors in *Bp* are controlled by the two-component or quorum sensing systems, indicating that *Bp* regulates its virulence systems by closely monitoring environmental cues (Jones et al., 1997; Ulrich et al., 2004; Valade et al., 2004; Horton et al., 2013; Schaefers, 2020). Bacterial gene regulation in response to environmental cues is vital for a successful transition from environmental living to intracellular infection (Ernst et al., 1999).

*BP1026B_I0091/BPSL0097* (*sap1*) encodes a surface attachment protein that is expressed within the host cell endocytic vesicles during *Bp* infection (Heacock-Kang et al., 2021). Sap1 is a virulence factor required for the complete pathogenesis of *Bp* infecting cell lines and BALB/c mice, in particular contributing to the initial attachment stage of the infection (Heacock-Kang et al., 2021). Although *sap1* has been characterized as a significant virulence determinant with a unique gene expression pattern during *Bp* infection, the transcriptional control of *sap1* has yet to be identified. In this study, we used a high-throughput transposon mutagenesis screen coupled with Tn-Seq to identify potential regulators of *sap1*. We identified BP1026B_II1046 as a transcriptional regulator that activates the *sap1* gene and designated it as SapR. Furthermore, we used RNA-Seq to characterize the SapR associated transcriptional network, providing insights into its role during *Bp* infection.

## Materials and methods

### Bacterial strains and growth conditions

All manipulations of wildtype *Bp* were carried out in CDC approved and registered BSL-3 facilities at the University of Hawaiʻi at Mānoa (UHM) John A. Burns School of Medicine (JABSOM) and experiments with the select agent were performed in accordance with the recommended BSL-3 practices (Richmond and McKinney, 2007). Select-agent excluded *Bp* 1026b ∆*asd* strain and *Bp*82 strain (Propst et al., 2010) were manipulated in the BSL-2 laboratory at the UHM. The derivative of *Escherichia coli* EPmax10B (Bio-Rad) containing *lacI*^q^ and *pir* [Lab ID: E1869 (Norris et al., 2009)] were routinely used as a cloning strain. The *E. coli* conjugal and suicidal strain HPS1-*pir*-Δ*asd*-*mob-*Km-Δ*pro* (Lab ID: E463 (Kang et al., 2009)) was used for the mobilization of plasmid containing *oriT*-origin into *B. pseudomallei* 1026b ∆*asd* [B0011 (Norris et al., 2011)] strain.

Growth of *E. coli* Δ*asd* strains was carried out as previously described with 100 μg/ml of diaminopimelic acid (DAP; Sigma) supplied (Norris et al., 2009). Luria-Bertani (LB) medium (Difco) was generally used to culture all the bacteria. Wildtype *Bp* strain 1026b was cultured in LB while the B0011 strain (Norris et al., 2009) was cultured in LB with 200 μg/ml diaminopimelic acid (LB + DAP200) or in minimal glucose (MG) media (1 × M9 minimal medium plus 20 mM glucose) containing 1 mM lysine, 1 mM methionine, 1 mM threonine, and 200 μg/ml DAP (3AA + DAP200). Diaminopimelic acid was prepared in 1 M NaOH as a 100 mg/ml stock and was used when necessary as described previously (Barrett et al., 2008). *Bp*82 (Propst et al., 2010) is a Δ*purM* derivative of *Bp* 1026b, and is an adenine and thiamine auxotroph. To culture *Bp*82, LB was supplemented with 0.6 mM adenine (LB + Ade) and M9-glucose medium was supplemented with both 0.6 mM adenine and 0.0005% thiamine (Propst et al., 2010). Merodiploids arising from chromosomal integration of the suicidal plasmid are resolved by utilization of the counter selectable marker *pheS* and *sacB*. 0.2% (*w*/*v*) *p*-chlorophenylalanine (cPhe; dl-4-chlorophenylalanine; Acros Organics) and 15% sucrose were used in *Bp*, respectively. LB + 1,000 μg/ml kanamycin was used for purifying the 1026b::T24 insertion mutants. All bacterial growth was carried out at 37°C and shaken cultures were maintained at 225 rpm.

### Cell line and culture conditions

Murine macrophage cell RAW264.7 was cultured in a humidified CO_2_ incubator at 37°C with 5% CO_2._ Dulbecco’s modified Eagle’s medium (DMEM) containing 4,500 mg/l glucose with 4 mM L-glutamine was used for culturing cell lines. All culture media were supplemented with 10% (*v*/*v*) heat-inactivated fetal bovine serum (FBS; HyClone). Gibco 100× antibiotic/antimycotic (AntiAnti) was added at a 1 × working concentration (containing 100 U/ml of penicillin, 100 μg/ml of streptomycin, and 250 ng/ml of amphotericin B) to prevent contamination by various bacteria and fungi. RAW264.7 cells were maintained at 60–80% confluency and were passaged by scrapping cells from the Corning^™^ T-75 flask. The passage numbers of cell lines were carefully recorded. CellBIND multi-well plates were used for cell infection assays.

### Molecular methods and reagents

Oligonucleotides were synthesized through Integrated DNA Technology. Unless otherwise indicated, all restriction enzymes, deoxynucleoside triphosphates, T4 DNA polymerase, T4 polynucleotide kinase, and T4 DNA ligase were purchased from New England Biolabs and used according to the manufacturer’s suggestion. Plasmids and DNA gel bands were isolated using the Zyppy plasmid miniprep kit and Zymoclean gel DNA recovery kit, respectively (Zymo Research Corporation). *Escherichia coli* competent cells were prepared as previously described (Kang et al., 2007). DNA ligations, restriction endonuclease digestions, and agarose gel electrophoresis were performed according to standard techniques, and all other molecular techniques were carried out according to methods described previously by Sambrook and Russell (2001).

### SMART RACE promoter mapping for *sap1*

Prokaryotes promoter predictor from Berkeley Drosophila Genome Project (Reese, 2001)^1^ was used to predict *sap1* transcription start site. The transcript start sites of *Sap1* was then experimentally mapped using a previously described method by direct sequencing of SMART^™^ RACE (rapid amplification of cDNA end) products (Tabansky and Nurminsky, 2003). All reagents required for promoter mapping were supplied in the SMART RACE cDNA Amplification kit (Clontech, BD Biosciences). Briefly, *Bp* wildtype 1026b strain was cultured in 3 ml LB broth and harvested in exponential phase for RNA extraction. Total RNA was prepared using the TRIzol^®^ reagent (Invitrogen, Carlsbad, CA, United States), and isolated with an on-column DNase I treatment, using the Qiagen RNeasy plus mini kit. One μg of total RNA was used to generate RACE cDNA using the SMART RACE cDNA Amplification kit (Clontech, BD Biosciences) and following the manufacturer’s instructions. cDNA ends were amplified with SMART primer (5′-AAGCAGTGGTATCAACGCAGAGTACGCGGG-3′) and gene-specific primer (5′-GTCTTTCGACAGACATCC-3′) at a final concentration of 2 μM. The diluted cDNA was used as a template in a PCR with a second nested gene-specific primer (5′-CAGTTGAATCTCCTGCTGC-3′) and the SMART RACE primer (5′-AAGCAGTGGTATCAACGCAGAGT-3′). PCR products were purified from 2% agarose gel slices and sequenced with a third nested gene-specific primer (5′-AGCGATGCATCGCCGTTC-3′) at the University of Hawaii genomic core facility.

### Construct promoter-*lacZ* fusion strain in *Burkholderia pseudomallei* 1026b Δ*asd* (B0011)

The engineering of P*_sap1_*-*lacZ* reporter strain was performed as described below. About 200 bp from up and down stream flanking the mapped transcriptional start sit was amplified from *Bp* genomic DNA with primers 5′-GAATGCCGGGTACCGAATGCGCCTG-3′ and 5′-ATGTCGTGTCTCGAATTCGCTCGGGTC-3′. A 400 bp PCR product was digested with *Eco*RI and *Kpn*I and cloned into mini-Tn*7*-Km-lacZ vector digested with the same restriction enzymes. A reporter strain was constructed with a single-copy integration of a P*_sap1_*-*lacZ* fusion at a neutral *glmS* site in the chromosome of B0011 strain using the well-established mini-Tn*7* system (Koch et al., 2001; Choi et al., 2005; Kang et al., 2009) with the aid of a nonreplicating helper plasmid pTNS3 encoding the transposase(Kang et al., 2009). Briefly, plasmids mini-Tn*7*-Km-P*_sap1_*-*lacZ* and pTNS3 was isolated using Zyppy Plasmid Kit. These two plasmids were mixed at an equal molar ratio and transformed into B0011 strain *via* electroporation. To make electro-competent bacterial cells, B0011 was grown to exponential phase in LB + DAP200 broth and 1 ml bacterial culture was harvested by centrifugation and washed four times with sterile water to remove salts. After electroporation, B0011 cells were immediately resuspended in 2 ml fresh LB + DAP200 and recovered in a 37°C incubator shaking at 225 RPM for 2 h. Cells were plated on LB + DAP200 + Km500 to select for the successful integration of P*_sap1_*-*lacZ* fusion. The *B. pseudomallei* chromosomes contain three insertion sites downstream of three *glmS* genes (Choi et al., 2008). P*_sap1_*-*lacZ* fusion was PCR confirmed to insert once at the *glmS*2 site with primers 5′-ATTAGCTTACGACGCTACACCC-3′ and 5′-ACACGACGCAAGAGCGGAATC-3′. The insertion of mini-Tn*7* in bacterial chromosome is quite stable and the selective maintenance is not required (Norris et al., 2010). The reporter strain was purified three times before saving away in the -80°C freezer stocks due to the intrinsic stickiness of *Bp* cells.

### Transposon mutagenesis

Random mutagenesis was performed in the B0011 P*_sap1_*-*lacZ* fusion strain using the pBT20-*gat* plasmid containing the *Himar1* mariner transposon and its transposase-encoding *tnp* gene (Kulasekara et al., 2005). *Escherichia coli* strain E463 (HPS1-*pir*-Δ*asd*-*mob-*Km-Δ*pro*) was used as the conjugal donor to introduce transposon plasmid pBT20-*gat* into B0011 P*_sap1_*-*lacZ* fusion strain. Briefly, the donor and recipient were grown up to mid-exponential phase and the cells were harvested separately by centrifugation at 9000 × *g* for 1 min at room temperature and washed twice with 1 ml of fresh LB. The pellets were combined at approximately equal cell densities and gently resuspended in 50 ul of LB. The mixture was spotted onto a LB + DAP200 plate for conjugation at 37°C. After 4 h of conjugation, the bacteria were harvested and washed twice with 1 ×M9 buffer, then plated on 10×150 mm agar plates containing MG + DAP 200 μg/ml + 3AA (1 mM of methionine, lysine, and threonine) + 60 μg/ml X-gal + 0.4% glyphosate. Without adding amino acid proline, the donor strain is unable to grow. The recipient strain cannot grow with the presence of glyphosate unless it has pBT20-*gat* transposon inserted into the chromosomes. The plates were incubated at room temperature for at least 24 h to allow the color to fully develop after colonies were visible following incubation at 37^o^ C (Supplementary Figure S1). The white and dark blue colonies were picked and patched on the same agar to confirm β-galactosidase activity. A total of 230,000 transposon mutant colonies, approximately 40 × coverage of genome, were screened for the β-galactosidase activity. 205 colonies with higher (dark blue) and 220 colonies with lower (white) β-galactosidase activity were pooled separately for transposon junctions identification through Illumina^®^ deep-sequencing (Tn-seq).

### Modified Tn-seq

Genomic DNA (gDNA) of dark blue and white colony pools was purified with phenol-chloroform extraction and quantified *via* Nanodrop. Shearing buffer (40% glycerol + 10 mM Tris pH 8.0 + 1 mM EDTA) was used to resuspend 10 μg of gDNA sample to a total volume of 800 μl followed by nebulization. Briefly, the samples were each nebulized for 5 min at 35 p.s.i. of N_2_ and followed by adding 30 mg/ml of dextran sulfate dissolved in water to each sample to a final concentration of 0.2 μg/ml. One tenth the volume of 3 M sodium acetate was added and vortexed to mix well. To precipitate DNA, an equal volume of ice cold isopropanol was added to the sample and freeze at –80^o^ C for at least 30 min. The samples were centrifuged at 4°C at full speed for 20 min and washed with old 70% ethanol two times. Excessive ethanol was further removed by heated speed vacuum and DNA pellets were resuspended in 16 μl Qiagen EB buffer for Nanodrop quantification. The sheared DNA was end repaired with the NEB Next End Repair Kit according to the manufacturer’s protocol and purified with Qiagen QIAquick PCR Purification Kit following the protocol. Each sample was further divided into four samples and 3′ A-tailing was carried out with Taq polymerase at 70°C for 20 min. The adapters were designed following previously described method with modification for sequence specific to our transposon (Gallagher et al., 2011). Purified DNA samples were digested with *Xba*I overnight at 37°C to separate the two genome-transposon junctions. Two % TAE agarose gel was used to visualize the DNA samples and the DNA smears between 200 and 400 bp were excised and purified *via* QIAquick Gel Extraction Kit and eluted with 30 μl of EB buffer. Circularization of adaptor ligated gDNA and exonuclease digestion were performed as previously described using Ampligase heat stable ligase (Gallagher et al., 2011). The transposon ends were amplified with PCR and Tn-seq libraries were built with the NEBNext DNA library prep kit for Ilumina^®^ sequencing based on manufacturer’s protocols. Library samples were cleaned up with MinElute PCR purification Kit and eluted with EB buffer.

The sample was sequenced at the Genetics Core and Confocal Microscopy Facility at University of Hawaiʻi at Mānoa. The dark blue and the white colony sample pools received 3.2 and 4.5 million reads, respectively. Reads were mapped onto the *Bp* genome with Bowtie (Langmead et al., 2009) using the Galaxy suite (Blankenberg et al., 2001; Giardine et al., 2005; Goecks et al., 2010) to identify the transposon junctions.

### Engineering of 1026b *sapR*::T24 complement strain

The well-established mini-Tn*7* integration system was used to construct the 1026b *sapR*::T24 complement strain (Norris et al., 2010) in BSL-3 laboratory. Briefly, *sapR* and its predicted native promoter was cloned into mini-Tn*7*-*gat* plasmid, yielding mini-Tn*7*-*gat*-*sapR*. The helper plasmid pTNS3 encoding transposase was electroporated with mini-Tn*7*-*gat*-*sapR* into the 1026b *sapR*::T24 mutant. After recovering in LB for 2 h, cells were harvested by centrifugation in a bio-safety cabinet and washed twice with 1x M9 minimal salts buffer to remove trace nutrient from LB. Mutants contains the mini-Tn*7* insertion were selected for on MG + 0.4% GS plate. Colonies were purified on the same medium three times and insertion at one of the three *glmS* sites in the chromosome was verified by PCR as previously described (Kang et al., 2009). This extensive purification procedure is very important and highly recommended, because GS is bacteriostatic, rather than bactericidal, at this effective concentration. 1026b *sapR*::T24 mutant, *sapR*::T24 complement, and the wildtype strain were used in the RT-PCR validation study and RNA-seq analysis.

### Real-time TaqMan RT-PCR analysis

We used our previously published primers and probe for *sap1* in this real-time PCR experiment (Heacock-Kang et al., 2021). The primers and probe were designed using Integrated DNA Technologies Primer Quest software^2^ following the criteria (Son et al., 2007) that were mentioned below. Briefly, the amplicon size should range from 60 to 100 bp and the primer melting temperatures were designed for 62°C, when more one primer pair were designed a melting temperature difference of less than 4°C for each pair would be recommended. The probe melting temperatures were designed to be 5–10°C higher than those for the corresponding primer pairs. To eliminate the possibility of nonspecific binding, primer and probe sequences were also subjected to BLASTN analysis against the *B. pseudomallei* wildtype 1026b genome, and the primers were also ensured to had no significant complementarity at the 3′ end and the probe to had no significant complementarity at the 5′ end to nonspecific locations on *Bp* 1026b genome.

The same preparation of RNA as described above for high throughput RNA-Seq experiment was used for real-time RT-PCR. Three μg of RNA was used for cDNA synthesis with random hexamer and SuperScript III (Invitrogen) per manufacturer’s protocol. To ensure no genomic DNA contamination was carried over from the RNA extraction, a control lacking reverse transcriptase was included for cDNA synthesis. The final cDNA product was dissolved in 1,200 μl RNase and DNase free water. In order to yield more accurate normalization analysis (Vandesompele et al., 2002) and control for variations between runs, three housekeeping genes (Heacock-Kang et al., 2021) (BPSL0602, BPSL2502, and BPSS2061 express consistently across all conditions) were chosen to be amplified at the same time with *sap1* on one 96-well plate. The primers set to amplify *sap1* are forward_5′-CATCGTTGCATTTTCGG-3′, reverse_ 5′-GGAATCCAGATTGTCTTTCG-3′, and probe_/56-FAM/TTCGTCAGTTGAATCTCCTGC/3BHQ_1/. Briefly, master mixtures were made and aliquoted into sub mixtures for each gene assayed. The final volume of each real-time PCR mixture is 25 μl containing 10 μl of cDNA, 12.5 μl of iQ Supermix (Bio- Rad), 120 nM of each primer, and 12 nM probe. Real-time PCR was performed with an iCycler iQ (Bio-Rad) using the following protocol: denaturation at 95°C for 10 min and then 55 cycles of amplification and quantification at 95°C for 20 s and at 65°C for 45 s. For each gene, reactions with amplification efficiency variant within 5% are subjected to data analysis. Real-time PCR fold changes were calculated using the amplification plot method and the available macro functions for data analyses of real-time PCR (DART-PCR; Peirson et al., 2003).

### Construction of *Bp*82 Δ*sapR* mutant and complement strain

Deletion mutation of *sapR* gene in *Bp*82 was made by allelic-replacement method based on the concept of the double homologous recombination as previously described (Barrett et al., 2008) with minor modification. *Bp*82 was grown in LB + Ade to exponential phase and harvested by centrifugation. Electro-competent *Bp*82 cells were made as described above (Barrett et al., 2008). Briefly, cells were washed four times with sterile ddH_2_O for electroporation with the allelic-replacement and suicidal vector pEX18Km-*pheS*-Δ*sapR*-*gat*-*FRT2*. After 2–3 h recovery in fresh LB + Ade, the bacterial cells were plated onto MG + 0.4% GS Ade/Thi agar plate. Merodiploids arising from chromosomal integration of the suicide plasmid are resolved by utilization of the counter selectable marker *pheS*. Colonies were purified on MG + 0.4% GS + 0.2% cPhe + Ade/Thi agar plates to resolve the merodiploid state. Plates were incubated at 37°C for 48 h, colonies were patched with toothpicks onto both MG + 0.4% GS + 0.2% cPhe + Ade/Thi and LB + Ade + Km 500 μg/ml plates. Mutants unable to grow in the presence of kanamycin were purified on MG + 0.4% GS Ade/Thi. Single colonies were purified on MG + 0.4% GS Ade/Thi media. The same purification procedure was repeated three times ensuring no wildtype background carrying over. Further screening and confirmation of mutants were performed by PCR using oligonucleotides pairs 5′-TGTCGATGCCGTCGAAG-3′/5′-CACATCCTGATGTACAAGCGGATCA-3′ (upstream junction) and 5′-GGCATCGAGAGTGTCATAC-3′/5′-CGTTGATGGGCTTGACCTCGATC-3′ (downstream junction) to amplify the chromosome and vector junctions. Primers 5′-CTGTCGATGCCGTCGAAG-3′ and 5′-GGCATCGAGAGTGTCATAC-3′ annealed to the upstream region of the chromosome outside of the region cloned for allelic replacement and the downstream region, respectively.

An unmarked strain was required to be constructed before engineering the complement strain. The *gat*-*FRT2* marker excision was performed using a previously described protocol (Choi et al., 2008) with minor modifications. Briefly, Δ*sapR* mutant was electroporated with 200 ng of pFlpe4, a curable Flp recombinase-expressing plasmid. Hundred μl aliquot of 1 ml recovery culture was plated on LB + Ade + Km500 + 0.2% l-rhamnose plate and incubated at 30°C for 48 h or until colonies were grown up. Single colonies were picked and patched on the same media to incubate at 30°C to ensure full excision. Fully-grown patches were streaked out on LB + Ade plate for single colony isolation and pFlpe4 plasmid was cured by incubating at 42°C. The successful excision of *gat* marker was confirmed by PCR and no growth on MG + 0.4% GS Ade/Thi plate. The well-established mini-Tn7 integration system was used to construct the *Bp*82 Δ*sapR* complement strain (Norris et al., 2010) in BSL-2 laboratory as described above. *Bp*82 Δ*sapR* mutant and Δ*sapR*/ comp were used in the mammalian cell infection assay.

### Growth analysis

*Bp82*, *Bp*82 Δ*sap1*, *Bp*82 Δ*sapR*, and *Bp*82 Δ*sapR* complement strains were grown up overnight in LB + Ade broth in a shaking incubator at 37°C. The bacteria were harvested and diluted 100 times in 250 μl fresh LB + Ade and placed in a 96-well plate. This experiment was conducted in triplicate. Growth analysis was carried out in the BioTek EL × 808IU plate reader at 37°C with constant shaking and OD_600_ measurement was recorded every 30 min. Data was analyzed and plotted in Prism.

### Construction of inducible broad-host range vector pBLAC

pBLAC is a derivative of pMLBAD, a low-copy broad-host-range plasmid (Lefebre and Valvano, 2002) that can replicate in *Bp* (Barrett et al., 2008). The *sapR* gene is placed immediately downstream of a *lac* operator and the expression of *sapR* can be induced by IPTG. pBLAC was constructed by replacing the *E. coli araBAD* arabinose-inducible promoter and *araC*, transcriptional regulator gene, of pMLBAD (Lefebre and Valvano, 2002) with *lac* promoter and *lacI-*encoded Lac repressor of mini-Tn*7*-*lacZ* that has been routinely used in our lab for *Bp* wildtype 1026b strain gene induction experiments. pBLAC was designed to be used for *sapR* induction study in *Bp*82 strains and its usage can be extended to *Burkholderia thailandensis* (*Bt*), the experimental surrogate of *Bp* that is manipulated in a BSL-1 laboratory. New vector pBLAC carries the backbone sequence derived from broad-host-range vector pMLBAD, including *mob*, gene required for conjugal transfer of plasmid; *rep*, replication protein gene; ori, origin of replication; the *dhfr*, dihydrofolate reductase gene encoding trimethoprim resistance; rrnB, transcriptional terminator; MCS, multiple cloning site. *SapR* cloned into the MCS was transcribed from the lac promoter and regulated by Lac repressor. Trimethoprim (Tp) 150 μg/ml was used in media to maintain the plasmid in *Bp*82 strains.

### Attachment assay

RAW264.7 macrophage cells were cultured in the T-75 corning flask to confluence and washed with pre-warmed sterile 1 x PBS twice. Then, cells were scraped off from the flask and seeded in 250 μl of DMEM without AntiAnti at 7 × 10^4^ cells per well into 48-well Corning CellBIND culture plates. Cells were allowed to attach overnight before the infection study. *Bp*82 strain grown in LB medium + Ade overnight at 37°C was used to infect RAW264.7 macrophages at MOI of 10:1 in the 48-well CellBIND plates. Bacteria were diluted in 1 × PBS for plating and numeration to obtain the initial infecting bacteria number. At 1 h post infection, DMEM containing unattached bacteria were removed and the cell monolayers were washed three times with pre-warmed sterile 1 × PBS. Monolayers were lysed with 500 μl 1 × PBS + 0.2% Triton X-100, and serial diluted in 1 × PBS. Fifty μl of 10^0^ (undiluted lysate), 10^−1^, 10^−2^, 10^−3^ dilutions were plated onto LB + Ade 12-well agar plates and colonies were enumerated after 48 h incubation at 37°C as the attached bacteria number. The attachment efficiency was determined by dividing the attached number by the initial infecting bacteria number. The experiment was carried out in triplicate and the numbers represent the average of all three replicates with the error bars representing the SEM. The student *t*-test was used to determine the statistical significance between attachment efficiencies of the different strains.

### Invasion and intracellular replication assay

Intracellular replication assay was carried out as previously described (Norris et al., 2011) with slight modifications. Briefly, RAW264.7 murine macrophage cells were infected with *Bp82*, mutant and complement strains at an MOI of 10:1 in an aminoglycoside protection assay. RAW264.7 cells were cultured and seeded into a 48-well Corning CellBIND culture plates as described above. After allowing the infection to progress for 1 h, the medium containing bacteria was removed, and the monolayers were washed three times with 1 × PBS to remove any unattached bacteria. Fresh DMEM + Ade with 1,000 μg/ml each of amikacin and kanamycin was added to the monolayers to kill any non-internalized bacteria and inhibit extracellular bacterial growth for the remainder of the assay. During the assay, medium was removed from the wells at 2, 8, and 24 hpi (hours post infection), and the infected cell monolayers were washed, lysed and diluted as described above. Bacteria colonies were numerated after 48 h incubation at 37°C, and data was calculated and plotted in Prism. All the assays were performed in triplicate, and the SEM were calculated for each.

### Transcriptomic analysis through RNA-Seq

*Burkholderia pseudomallei* wildtype strain1026b and *sapR*::T24 insertion mutant, were grown overnight in 3 ml LB broth, subcultured to grow to mid exponential phase for RNA extraction. Total RNA from 12 ml culture of each strain was isolated using TRIzol^®^ reagent and purify through QIAGEN RNeasy plus mini kit according to the manufacturer’s instructions. Sequencing was performed by the Tufts University Core Facility Genomics on the Illumina HiSeq 2500 platform after rRNA depletion and library generation. More than 20 million reads with > 96% alignment rate to *Bp* genome were generated for each triplicate sample from each strain. The fastq files were uploaded to Rockhopper tool (McClure et al., 2013) designed specifically for analysis of bacterial RNA-Seq data. The normalized expression values generated in the Rockhopper analysis, in which the *sapR* mutant was compared to the wild type control, were used for the differential gene expression analysis. Genes with a false discovery rate (*q*-value) of < 0.01 were considered to be significantly differentially expressed and were designated for further analysis. A subset of these significantly differentially expressed genes with log_2_FC ≤ −1 and ≥ 1 were used for Clusters of Orthologous Groups of proteins (COG) functional analysis and subcellular localization analysis for their gene products. The subcellular localization was computationally predicted through PSORTb V3.0 (Yu et al., 2010). The list of predicted gene across the whole genome was downloaded from Burkholderia.com to use in the analysis. Graphs were generated in Prism. Differentially regulated gene clusters (*q*-value < 0.01) identified from RNA-Seq were mapped along two *Bp* chromosomes using WoPPER web server (Puccio et al., 2017) to visualize their physical localizations.

## Results

### Mapping the regulatory region of *sap1*

**Figure 1:**
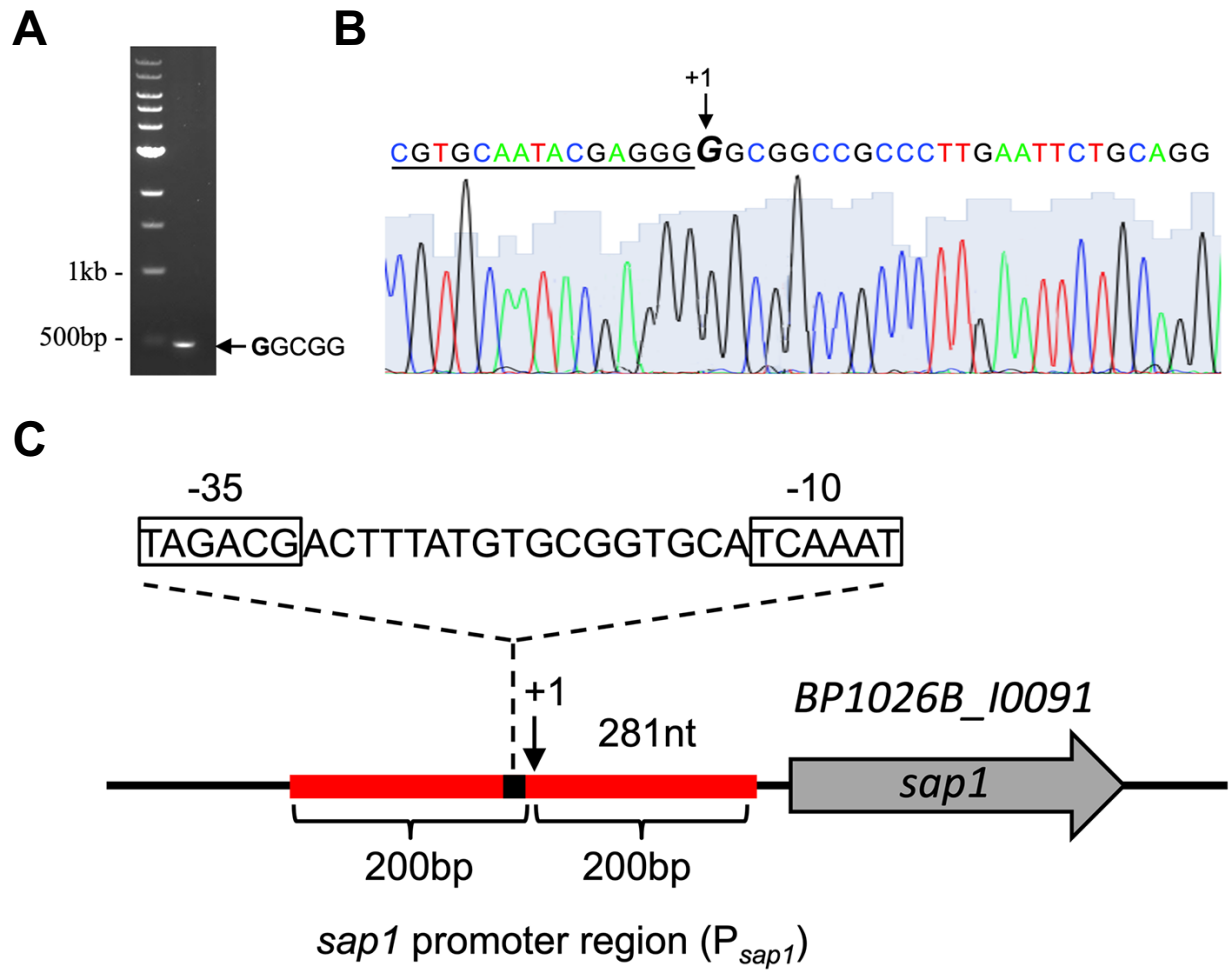
SMART RACE mapping identifies the transcriptional start site for *sap1*. **(A)** One SMART product was observed after PCR amplification of the cDNA with SMART primer and a second nested gene-specific primer. **(B)** The *sap1* transcriptional start site (indicated by + 1 at the GGCGG sequence) was identified by sequencing the purified PCR product with a third nested gene-specific primer. The underlined nucleotides indicate the SMART primer sequence. **(C)** Schematic representation of *sap1* locus. The *sap1* transcript start site is at 281 bp upstream of its start codon; the sap1 promoter region, 200 bp flanking the predicted transcriptional start site (indicated by red line), was used to identify potential regulators of *sap1*; the sequences of predicted − 10 and − 35 elements were shown.

To find regulators of *sap1* using high-throughput transposon mutagenesis and deep sequencing, we must first determine the approximate *sap1* regulatory region by identifying its transcriptional start site. Using the neural network based promoter prediction server of the Berkeley Drosophila Genome Project (BDGP; Reese, 2001), we found two possible transcriptional start sites. The first possible transcriptional start site is 182 bp before the *sap1* start codon and has a confidence value of 0.81. The second possible transcriptional start site is 281 bp before the *sap1* start codon and has a confidence value of 1.00. Transcription of *sap1* could occur from one or both of these possible transcriptional start sites. To experimentally determine if one or both of these transcriptional start sites is used, we employed the well-established non-radioactive SMART RACE method (Tabansky and Nurminsky, 2003). As shown in Figure 1A, one SMART product approximately 500 bp in size was obtained. Sanger sequencing determined that the *sap1* transcriptional start site is 281 bp upstream of its start codon, confirming the high confidence prediction from BDGP (Figures 1B,C). From this information, we determined the promoter of *sap1* (P*_sap1_*) and identified the −10 and − 35 elements (Figure 1C). The *sap1* promoter region encompassing 200 bps flanking either side of the predicted *sap1* transcriptional start site was utilized to identify potential regulators of *sap1* (Figure 1C).

### Identification of BP1026B_II1046 as a potential regulator of *sap1*

**Figure 2:**
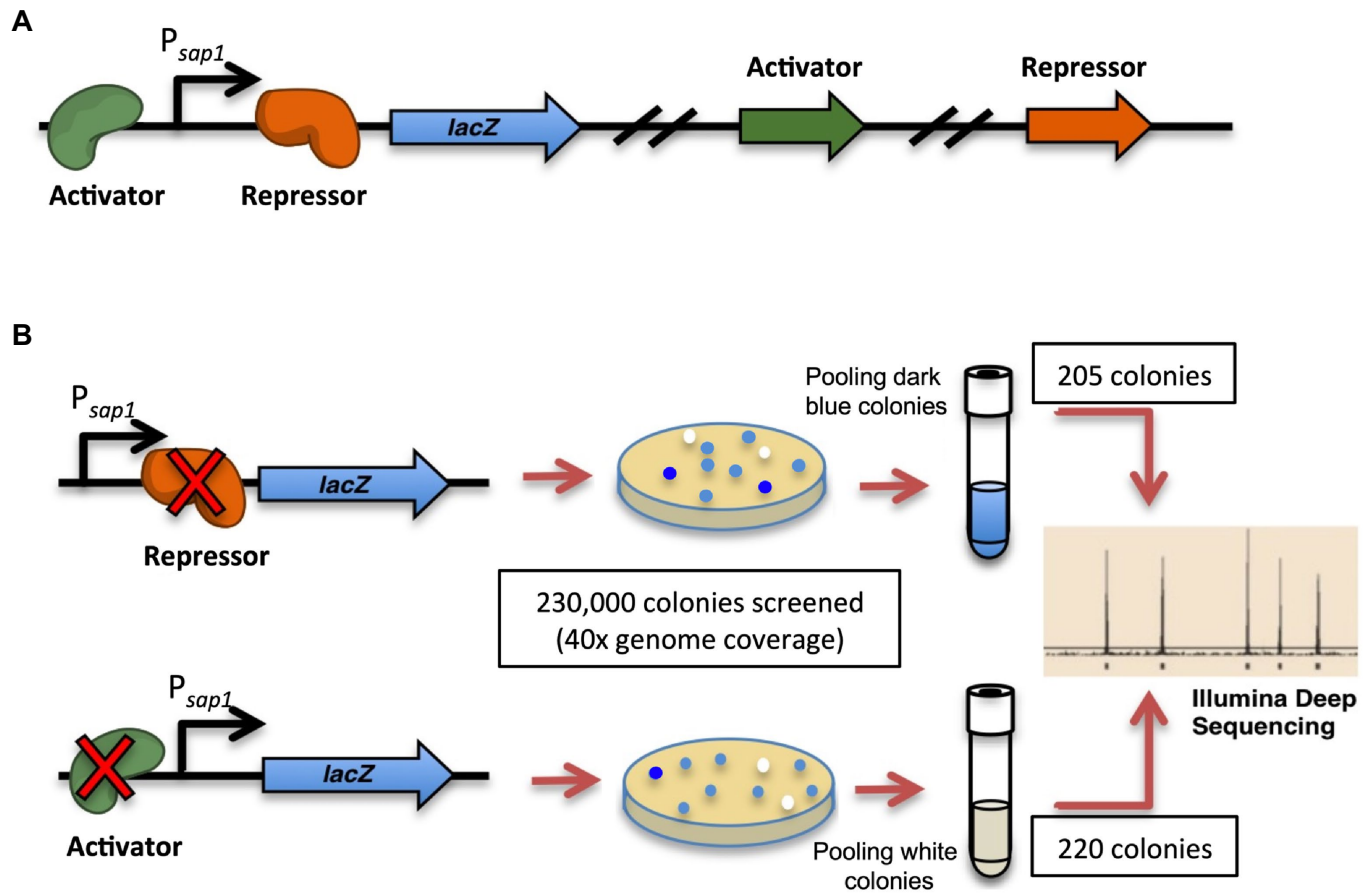
Strategy to identify potential *sap1* regulator(s): high-throughput transposon mutagenesis and Tn-Seq. **(A)** A reporter strain was generated with a single-copy integration of a P*_sap1_*-*lacZ* fusion at a neutral site on the chromosome of B0011 strain. **(B)** Random mutagenesis was performed on the reporter strain using mariner transposon pBT20-*gat*. A total of 230,000 transposon mutant colonies, approximately 40 × coverage of genome, were screened for their β-galactosidase activities. Colonies with higher (205) and lower (220) expression of β-galactosidase activity were pooled separately and the transposon junctions were identified by Tn-Seq.

We designed a high-throughput strategy to identify the regulator(s) for *sap1* using transposon mutagenesis of the select-agent exempt strain *Bp* 1026b Δ*asd* (B0011) and deep sequencing (Figure 2; Gallagher et al., 2011; Norris et al., 2011). A reporter strain was constructed with a single-copy integration of a P*_sap1_*-*lacZ* fusion at a neutral site on the chromosome of B0011 using the mini-Tn*7* system (Koch et al., 2001; Choi et al., 2005; Kang et al., 2009). Random mutagenesis was then performed on this B0011 reporter fusion strain, utilizing the mariner transposon on pBT20-*gat* (Kulasekara et al., 2005). To ensure comprehensive coverage of both chromosomes, a total of 230,000 transposon mutant colonies, approximately 40 × coverage of *Bp* genome, were screened for variations in β-galactosidase activity. Colonies with increased β-galactosidase activity would have dark blue color indicating that the transposon insertions disrupted genes that repress expression from the *sap1* promoter (Figure 2B). On the other hand, if a gene that activates expression from the *sap1* promoter was mutated, lower β-galactosidase activity would be observed, and colonies would be less blue than the basal level (Figure 2B). Several hundreds of both dark blue and white colonies were patched to ensure a consistent β-galactosidase activity pattern. Eventually, 205 dark blue colonies and 220 white colonies were pooled separately and the transposon junctions were identified by Tn-seq^49^. From the pool of white colonies and through this analysis, we identified BP1026B_II1046, a hypothetical protein with a DNA binding motif. BP1026B_III046 shows similarities to MurR/RipR DNA binding transcriptional regulators (COG1737) with an N-terminal helix-turn-helix like HTH_6 domain (Pfam: PF01418) and a C-terminal a SIS (Sugar ISomerase) domain (cd05013), which is found in many phosphosugar isomerases and phosphosugar binding proteins (Sørensen and Hove-Jensen, 1996; Finn et al., 2016; Lu et al., 2020). In addition, both Phyre2 (Kelley et al., 2015) and I-TASSER (Zhang, 2009; Roy et al., 2012; Yang and Zhang, 2015) predicted that BP1026B_II1046 was homologous to *Vibrio vulnificus* NanR further indicating that BP1026B_II1046 is a regulator. We, therefore, designated *BP1026B_II1046* as *sapR* for the remainder of this study.

We used qRT-PCR to verify SapR regulatory activity on *sap1* (Table 1). RNA was isolated from *Bp* 1026b wildtype strain and a *sapR* mutant strain from the *Bp* 1026b T24 transposon mutant library (Borlee et al., 2017). In the absence of SapR, the *sap1* gene was down-regulated with a log_2_FC (fold change) of −0.78 compared to wildtype *Bp* 1026b, consistent with the lower β-galactosidase activity in our initial transposon mutagenesis screening (Table 1). We performed beta-gal assay (Son et al., 2008) in the P*_sap1_*-lacZ reporter strains of *Bp*82 and *Bp*82 Δ*sapR* mutant to further quantify the regulatory activity of SapR. The beta-galactosidase activity was significantly reduced in the Δ*sapR* mutant, and the reduction level agreed with our RNA-seq and RT-PCR results (Supplementary Figure S2). Additionally, from our previous *Bp* single cell ‘TRANSITomic’ analysis that revealed dynamic gene-expression flux as *Bp* transit through RAW264.7 macrophage at defined stages: vacuole entry; cytoplasmic escape and replication; and membrane protrusion (Heacock-Kang et al., 2021), we identified that both *sap1* and *sapR* were up-regulated in the endocytic vesicle. This finding suggests a coordinated expression of *sap1* and *sapR* during intracellular infection (Supplementary Figure S4). Together these data indicate that SapR is an activator of the *sap1* gene.

**Table 1:** Summary of experimental data indicating that SapR positively regulates *sap1*.

Tn-seq^1^		Mutagenesis	Log_2_ FC (M/W) of *sap1* regulon	
Gene ID	Function	β-galactosidase activity	Real-time PCR^2^	RNA-seq^3^
BP1026B_II1046	Predicted to be a MurR/RipR family transcriptional regulator	Reduction^4^	−0.78	−0.56

1 Genes identified through Tn-seq from white and dark blue colony pools.

2 Real-time PCR to measure *sap1* regulon expression level quantitatively, and the value of p was calculated to be 0.0009 via a one-way t-test.

3 Log_2_ FC (expression RPKM of *sap1* from mutant vs expression RPKM of *sap1* from wildtype). Next-generation sequencing to measure *sap1* regulon expression level quantitatively, and the q-value was determined to be 9.56E-93.

4 White colony pool that mutation repressed P_sap1_-lacZ appearing with lower β-galactosidase activity.

### SapR is important for regulating *Burkholderia pseudomallei* attachment to RAW264.7 murine macrophage cells

**Figure 3:**
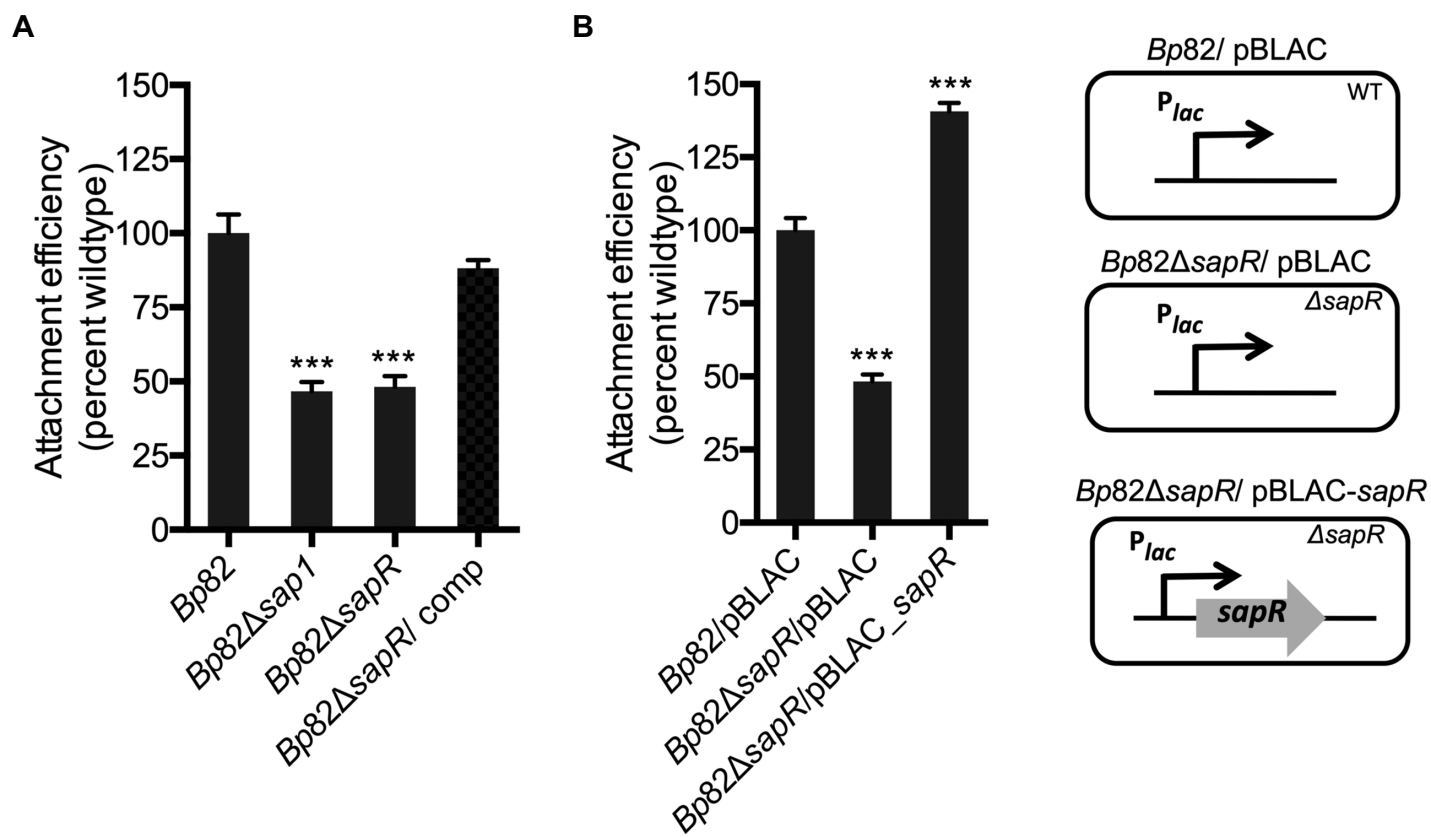
SapR is required to activate *sap1* for attaching to host cells. Attachment assays on RAW264.7 macrophage monolayers were performed to further validate SapR’s role as an activator for *sap1*. **(A)** The *Bp*82 Δ*sapR* mutant exhibited significant attachment deficiency to RAW264.7 cells, comparing to the *Bp82* strain. This deficiency is comparable to the defect in attachment of the *Bp*82 Δ*sap1* strain. Complement with a single copy of *sapR* restored the impaired attachment to wildtype level. **(B)** *Bp*82 Δ*sapR* mutant/pBLAC displayed drastic defect in its attachment ability. Whereas the constitutive expression of *sapR* in Δ*sapR* mutant/pBLAC-*sapR* by inducing the culture with 1 mM IPTG increased bacterial attachment significantly. Data in bar graphs represent means ± s.e.m. and analyzed *via* unpaired *t*-test. ^***^p < 0.001.

To further validate the SapR regulatory function of *sap1,* attachment assays that measure the efficiency of bacterial adherence to the host cell surface were carried out, using the select-agent excluded *Bp*82 strain derived from wildtype strain 1026b. RAW264.7 murine macrophages were infected with strains *Bp82*, *Bp*82 Δ*sapR*, *Bp*82 Δ*sapR-attTn7::sapR* complement (*Bp*82 Δ*sapR/*comp), and *Bp*82 Δ*sap1* at an MOI of 10:1. The Δ*sapR* mutant was significantly defective in attachment to RAW264.7 monolayers, showing 48.16% of *Bp82* attachment ability (Figure 3A). This is comparable to the attachment defect of the Δ*sap1* mutant exhibiting 46.72% wildtype attachment (Figure 3A). This result shows that the Δ*sapR* mutant has a similar phenotype to the Δ*sap1* mutant during host cell infection and suggests that SapR and Sap1 act in a similar pathway. The attachment defect of the Δ*sapR* mutant was restored by single copy complementation of the *sapR* gene.

In addition, we over expressed SapR *in trans* to compare the attachment efficiencies with *Bp*82 and *Bp*82 Δ*sapR* mutant. RAW264.7 murine macrophages were infected with *Bp82*/pBLAC, *Bp*82 Δ*sapR*/pBLAC, and the over-expressed *sapR* strain *Bp*82 Δ*sapR*/pBLAC-*sapR* all under IPTG induction (Figure 3B). The strain Δ*sapR* /pBLAC-*sapR* that over expresses SapR demonstrated a significantly increased ability to attachment to RAW264.7 cells, at approximately 140% of *Bp*82 (Figure 3B). This observation that increased expression of *sapR* leads to increased attachment provides further evidence that SapR is an activator of *sap1*. Taken together, these data suggest that both SapR and Sap1 have roles in the host cell attachment process.

### The Δ*sapR* mutant has reduced intracellular replication ability

**Figure 4:**
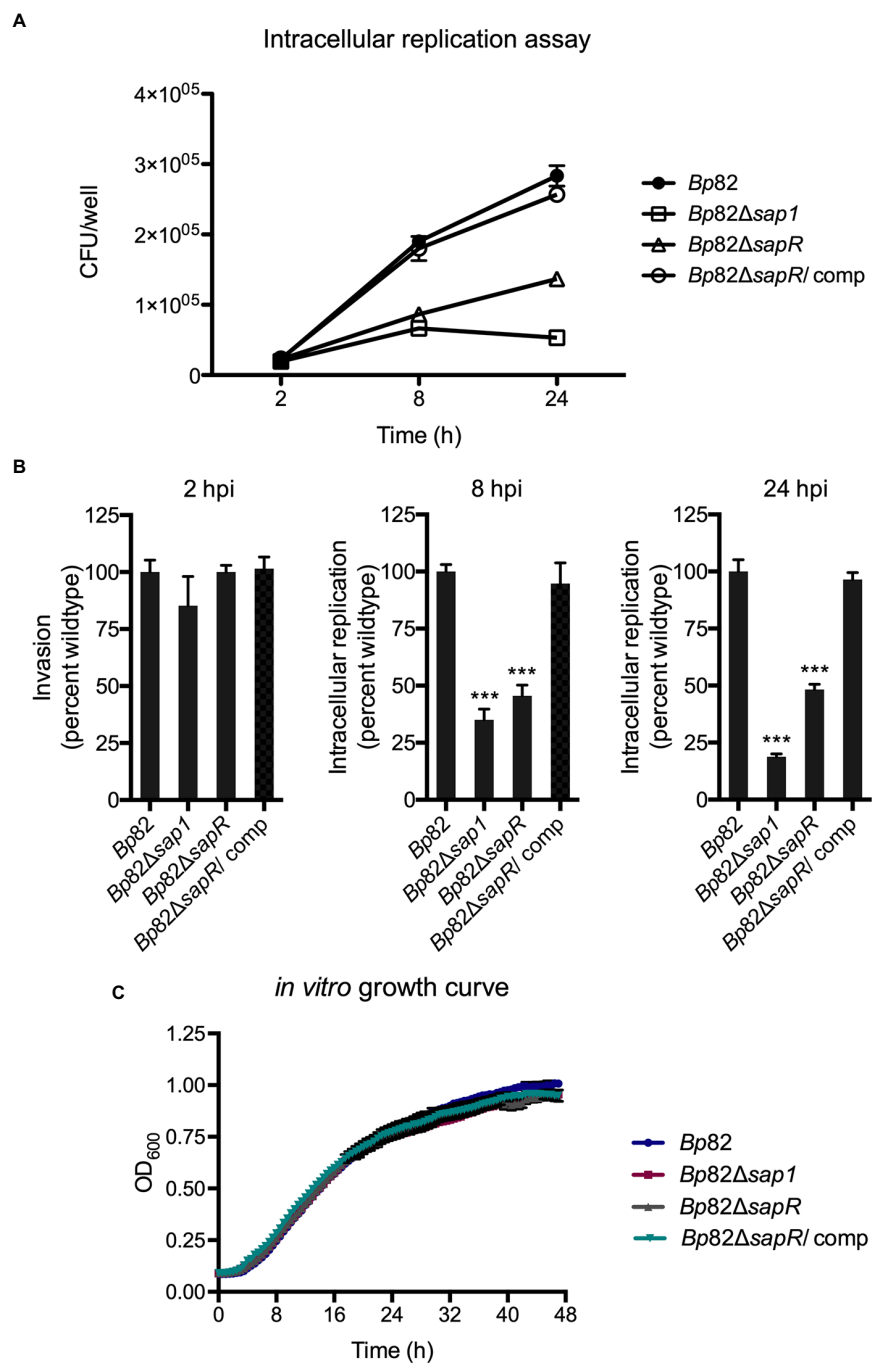
The Δ*sapR* mutant is highly defective during intracellular replication. **(A)** *Bp82*, Δ*sap1*, Δ*sapR*, and Δ*sapR*/comp strains were analyzed for their ability to replicate in RAW264.7 murine macrophages at initial MOI of 10:1. CFU/well of each strain was numerated at 2, 8, and 24 h post infection (hpi). **(B)** The percent wildtype were calculated and the averages of triplicates were presented with error bars representing SEM. The Δ*sap1* and Δ*sapR* mutants displayed significant defect in replication at 8 and 24 hpi compared to the *Bp82* strain. Data in bar graphs represent means ± s.e.m. and analyzed *via* unpaired *t*-test. ^***^p < 0.001. **(C)** *In vitro* growth analysis of *Bp*82, Δ*sap1*, Δ*sapR*, and Δ*sapR*/comp in LB + Ade liquid media. Mutants show no *in vitro* growth defects compared to wildtype strain while growing under the same conditions.

To investigate if the attachment deficiencies of the Δ*sapR* mutant lead to subsequent decreased in intracellular replication, an intracellular replication assay was carried out in RAW264.7 macrophage cells. RAW264.7 cells were infected with an MOI of 10:1 with *Bp82*, *Bp*82 Δ*sapR, Bp*82 Δ*sapR-attTn7::sapR* complement (*Bp*82 Δ*sapR/*comp), and *Bp*82 Δ*sap1* strains (Figure 4A). At 2 h post infection (hpi), no significant difference was observed initially amongst all the strains indicating a similar number of bacteria were uptake by macrophage cells. As the infection progressed, the intracellular replication capability of *Bp*82 Δ*sapR* was significantly impaired, replicating at 45% of *Bp*82 at 8 hpi and 48% of *Bp*82 at 24 hpi (Figure 4B). The defect of the Δ*sapR* mutant was restored by single copy complementation of the *sapR* gene (Figures 4A,B). These results are similar in the *Bp*82 Δ*sap1* mutant showing defects at 8 h and 24 hpi, replicating at 35 and 18 percent of *Bp*82 (Figure 4B). *In vitro* growth analysis showed that these mutants have similar growth patterns to wildtype and complement strains (Figure 4C). Taken together, the results indicate that the defect in intracellular replication of the *Bp*82 Δ*sapR* mutant is due to impaired pathogenesis rather than defect in *in vitro* fitness.

### SapR regulates membrane associated virulence factors, peptidoglycan, lipid, and nitrogen metabolism

We have shown that SapR controls the expression of *sap1* and that there is a functional correlation between these two proteins. To further understand the role of SapR in the context of *Bp* pathophysiology, we sought to determine the regulation network of SapR. We compared the transcriptome profile from *Bp* 1026b wildtype and 1026b *sapR*::T24 mutant strains growing under the same conditions using RNA-Seq analysis. The *sap1* gene was differentially expressed with a log_2_FC of −0.56 and *q*-value (the false discovery rate) of 9.56E-93, further confirming that SapR is a positive regulator of *sap1* (Table 1). To gain a better understanding of the SapR regulatory network, we did a further investigation on the role of SapR regulating membrane associate components which was summarized in Table 2. This table listed interesting findings of differentially expressed membrane associate components that are either known to associate with *Bp* pathogenesis or newly identified membrane-associated clusters from WoPPER analysis in our study.

**Table 2:** Genes regulated by SapR encoding products closely associated with cell envelope.

Putative annotation	Gene ID	Product description	Subcellular localization^a^	COG function prediction^a^	Log_2_ FC (M/WT)
*tssM-2*	BP1026B_II0111	Type VI secretion system (T6SS)	Cytoplasmic membrane	Intracellular trafficking, secretion, and vesicular transport	1
*tssM-6*	BP1026B_II2265	Type VI secretion system (T6SS)	Outer membrane	Intracellular trafficking, secretion, and vesicular transport	1
*bopA*	BP1026B_II1619	BopA protein (T3SS3)	Extracellular	NA	−0.98
*bipB*	BP1026B_II1628	BipB protein (T3SS3)	Extracellular	NA	−0.89
*bipC*	BP1026B_II1627	Cell invasion protein (T3SS3)	Periplasmic	NA	−0.98
					
	BP1026B_II1580	Anaerobically induced outer membrane protein	Periplasmic	Secondary metabolites biosynthesis, transport and catabolism	5.29
	BP1026B_II0774	Outer membrane porin protein	Outer membrane	Cell wall/membrane/envelope biogenesis	−1.32
	BP1026B_I0046	Outer membrane porin	Outer membrane	Cell wall/membrane/envelope biogenesis	1.00
	BP1026B_II0620	Outer membrane porin	Outer membrane	Cell wall/membrane/envelope biogenesis	1.58
	BP1026B_I3218	Carbohydrate porin	Outer membrane	Cell wall/membrane/envelope biogenesis	1.72
	BP1026B_II1369	Outer membrane protein TolC	Outer membrane	Cell wall/membrane/envelope biogenesis	1.64
*batA*	BP1026B_I1153	Outer membrane autotransporter	Outer membrane	Lipid transport and metabolism	−0.58
	BP1026B_II1040	ABC transporter ATP-binding protein	Cytoplasmic membrane	Amino acid transport and metabolism	−1.68
	BP1026B_II1041	ABC transporter ATP-binding protein	Cytoplasmic membrane	Amino acid transport and metabolism	−2.66
	BP1026B_II1042	Dipeptide transport system permease	Cytoplasmic membrane	Amino acid transport and metabolism	−2.46
	BP1026B_II1043	ABC transporter permease	Cytoplasmic membrane	Amino acid transport and metabolism	−2.58
	BP1026B_II1044	Periplasmic solute-binding protein	Periplasmic	Amino acid transport and metabolism	−3.46
	BP1026B_II1045	D-alanyl-D-alanine dipeptidase	Cytoplasmic	Cell wall/membrane/envelope biogenesis	−1.32
					
*narI-1*	BP1026B_I1015	Respiratory nitrate reductase subunit gamma	Cytoplasmic membrane	Energy production and conversion	1.39
*narJ-1*	BP1026B_I1016	Nitrate reductase subunit delta	Cytoplasmic	Energy production and conversion	1.22
*narH-1*	BP1026B_I1017	Nitrate reductase subunit beta	Unknown (multi-location sites)	Energy production and conversion	1.51
*narG-1*	BP1026B_I1018	Nitrate reductase subunit alpha	Cytoplasmic membrane	Energy production and conversion	1.80
*narK-2*^b^	BP1026B_I1019	Nitrate/nitrite transporter NarK	Cytoplasmic membranes	Inorganic ion transport and metabolism	2.36
*nark-1*^b^	BP1026B_I1020	Nitrate/nitrite transporter	Cytoplasmic membrane	Inorganic ion transport and metabolism	2.09
*nark*	BP1026B_II1220	Nitrate/nitrite transporter	Cytoplasmic membrane	Inorganic ion transport and metabolism	−0.32
*narI-2*	BP1026B_II1222	Respiratory nitrate reductase subunit gamma	Cytoplasmic membrane	Energy production and conversion	−1.18
*narJ-2*	BP1026B_II1223	Nitrate reductase 1 subunit delta	Cytoplasmic	Energy production and conversion	−1.32
*narH-2*	BP1026B_II1224	Nitrate reductase subunit beta	Cytoplasmic membrane	Energy production and conversion	−1.14
*narG-2*	BP1026B_II1225	Nitrate reductase subunit alpha	Cytoplasmic membrane	Energy production and conversion	−0.58

a Only genes with log_2_ FC ≤ –1 or ≥ 1 were shown in the subcellular localization analysis and COG function prediction analysis.

b Annotation from the Pseudomonas Genome Database; NA: COG function prediction not assigned.

**Figure 5:**
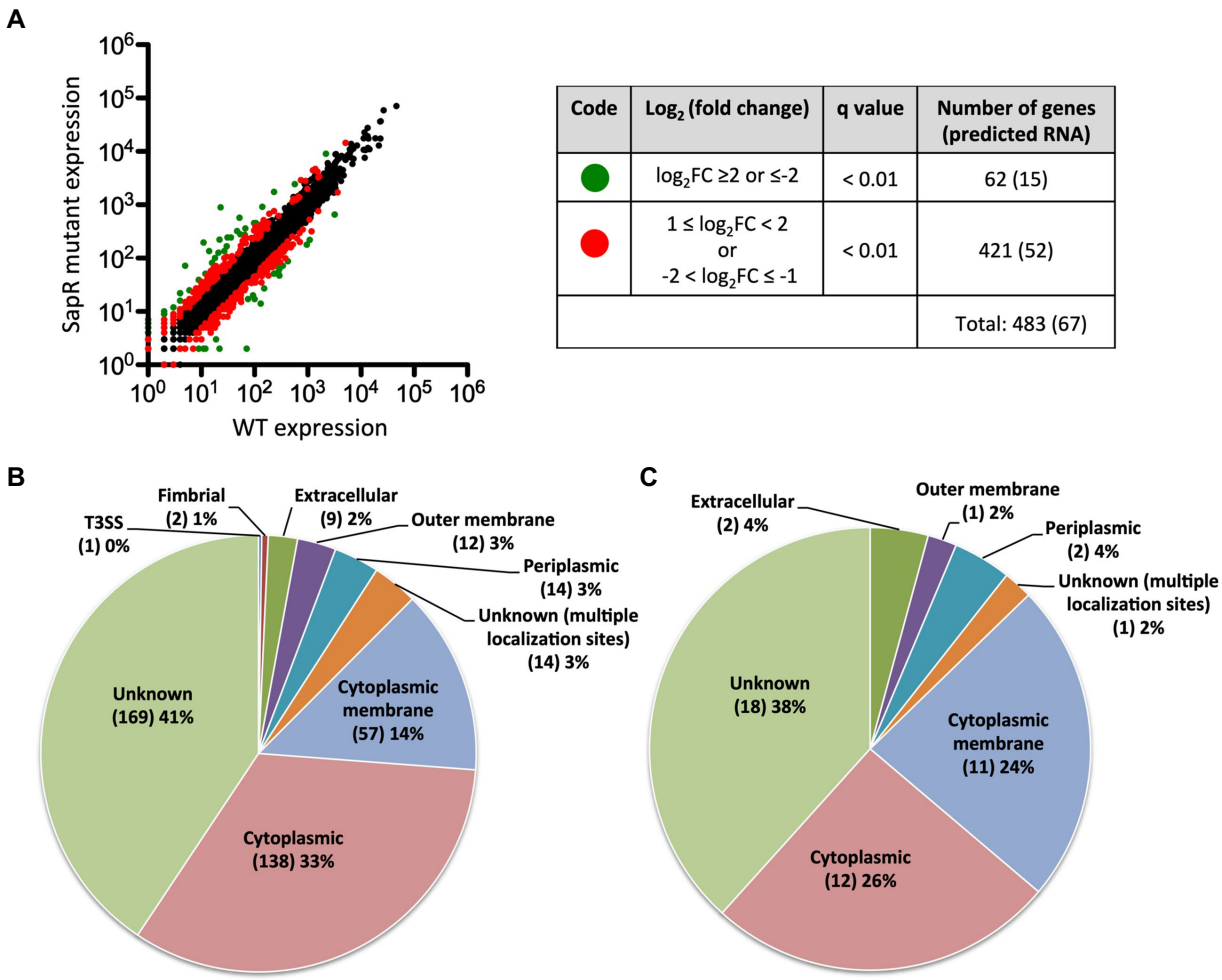
SapR differentially regulates genes in many subcellular locations. **(A)** Expression values generated from Rockhopper of all detected genes were used to create the plot. A total of 483 DEGs are highlighted. Sixty-two genes with a *q*-value < 0.01 and a log_2_FC ≤ −2 or ≥ 2 are highlighted in green, and 421 genes with 1 ≤ log_2_FC < 2 or − 2 < log_2_FC ≤ −1 are highlighted in red. Numbers of predicted RNAs shown in parentheses were not included in this analysis. **(B)** The 416 DEGs with a cut off of a log_2_FC ≤ −1 or ≥ 1 were further analyzed based on their assigned subcellular localization from Burkholderia.com. **(C)** A large enrichment was showed in proteins associated with cytoplasmic membrane while increasing the stringency to cut off of a log_2_FC ≤ −2 or ≥ 2. Number of genes in each category was presented in parenthesis followed by its percentage.

In total, 483 genes were regulated by SapR using a statistical cut off of q-value < 0.01 and a log_2_FC cut off of ≤ −1 or ≥ 1 (Supplementary Table S1; Figure 5A). Among these 483 differentially expressed genes (DEGs), there are 67 predicted RNA transcripts, and 416 annotated open reading frames. Of the 416 protein coding genes, 56% are assigned a subcellular location and 44% are unknown (Figure 5B). Some of these SapR-regulated proteins are associated with the cytoplasmic membrane (14%), periplasmic space (3%), outer membrane (3%) and extracellular space (2%; Figure 5B). Cytoplasmic membrane associated proteins showed a large enrichment to 24% when increasing the stringency to a log_2_FC cut off of ≤ −2 or ≥ 2 (Figure 5C). *BP1026B_I1015*, *BP1026B_I1020* and *BP1026B_II1220*, *BP1026B_II1225* associated with cell envelope for nitrogen metabolism were regulated by SapR with the mean of log_2_FC > 1 and < −1 (Table 2). SapR also regulated known virulence factors associated with pathogenesis. SapR regulates components of type III and type VI secretion systems (T3SS3 and T6SS). *BP1026B_II0111* (*tssM-2*) and *BP1026B_II2265* (*tssM-6*) encoding an inner membrane protein TssM essential for the function of a sub-complex of the T6SS. Both genes were significantly up-regulated in the *sapR* mutant with log_2_FC of 1 (Table 2).

BP1026B_II1619 (BopA), an effector of T3SS3 responsible for phagosome escape (Gong et al., 2011), and two translocators of T3SS3, BP1026B_II1628 (BipB) involved in stimulating cell-to-cell fusion and BP1026B_II1627 (BipC) bound to host F-actin to facilitate internalization of *Bp* into host cells (Kang et al., 2016; Vander Broek et al., 2017), were down-regulated in the *sapR* mutant. In addition, SapR regulates many outer membrane porins (BP1026B_II0620, BP1026B_II0774, BP1026B_II1369, BP1026B_I0046 and BP1026B_I3218) generally responsible for cellular permeability and antibiotic resistance (Choi and Lee, 2019). Furthermore, an autotransporter *BP1026B_I1153* (*batA*; Lafontaine et al., 2019) was down-regulated in the *sapR* mutant. We also observed altered expression of genes near the *sapR* gene, namely *BP1026B_II1040*–*BP1026B_II1045*, amongst the highest down-regulated genes in the SapR mutant (Table 2; Supplementary Figure S3). These include a dipeptidase, dipeptide and ABC transport systems, and a periplasmic binding protein that could be involved in peptidoglycan metabolism. The *sapR* mutant was complemented by inserting a copy of *sapR* back into the genome *via* the mini-Tn*7* system. This demonstrates that the transposon mutation of *sapR* is unlikely to cause polar effects on the adjacent genes. This evidence strongly suggests that SapR regulates many proteins associated with the cell envelope that are also tied to *Bp* pathogenesis.

**Figure 6:**
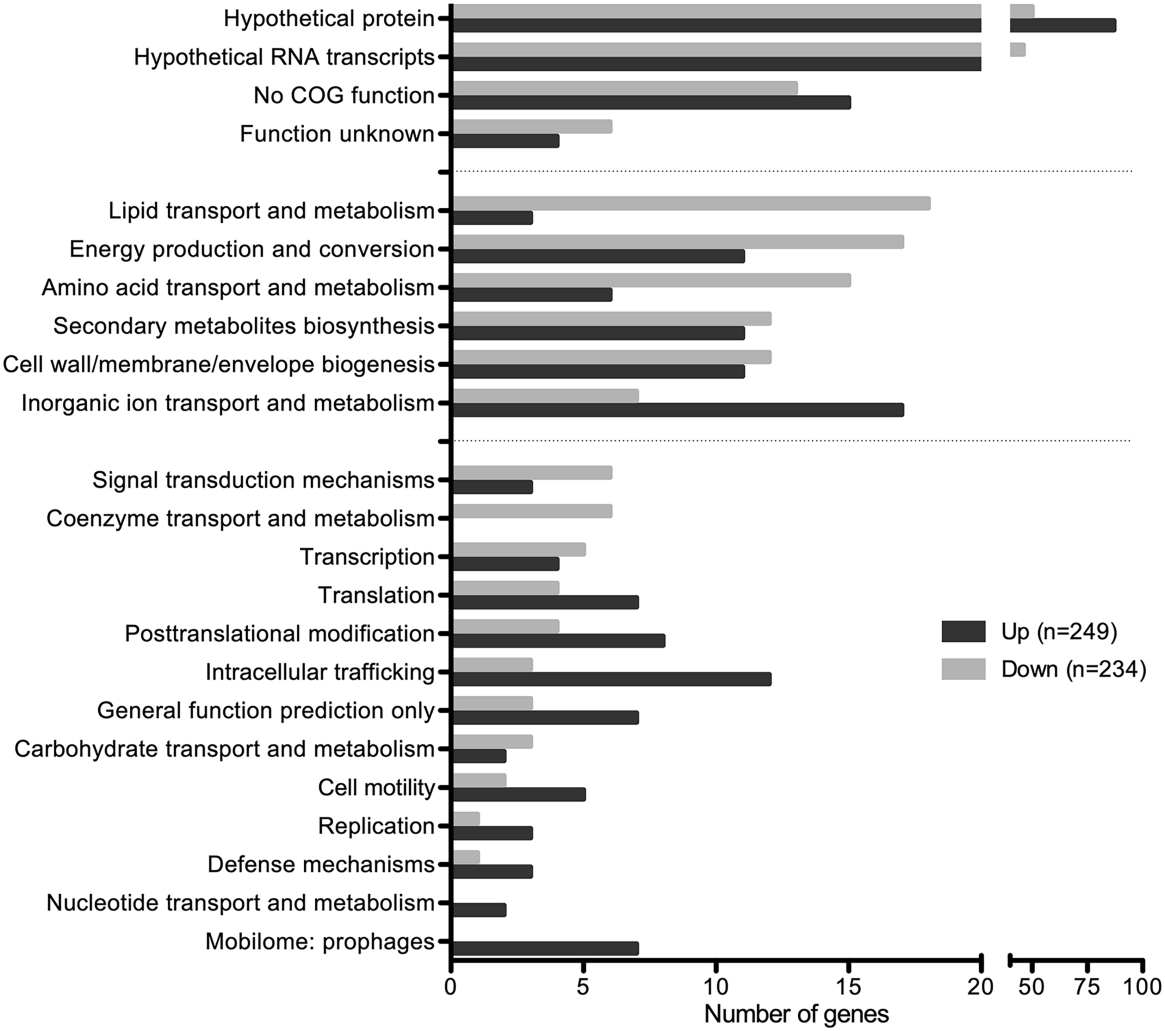
SapR disproportionally regulates hypothetical proteins. Differentially expressed genes (483) with statistical analysis (*q*-value < 0.01) combined with a cut off of log_2_ FC ≤ −1 or ≥ 1 were analyzed by the Clusters of Orthologous Genes (*COG*) functional predictions. Hypothetical proteins, proteins of unknown function, proteins with no COG prediction, or hypothetical RNA transcripts account for approximately 50% of DEGs. Among the rest, the top functional categories (29%) regulated by SapR are lipid transport and metabolism, energy production and conversion, amino acid transport and metabolism, cell wall/membrane/envelope biogenesis, secondary metabolites biosynthesis, transport and catabolism, and inorganic ion transport and metabolism.

Among the 483 DEGs, 249 were up regulated and 234 were down regulated (Figure 6; Supplementary Table S1). Approximately 50% of DEGs are classified as hypothetical proteins, proteins of unknown function, proteins with no COG prediction, or hypothetical RNA transcripts (Figure 6). The rest of DEGs encode core functions such as lipid, inorganic ion, carbohydrate, or amino acid transport and metabolism, energy production and conversion, secondary metabolites biosynthesis, and cell wall/membrane/envelope biogenesis (Figure 6). Among those functional categories, lipid transport and metabolism and energy production and conversion account for the largest proportions of the analyzed DEGs (Figure 6). This suggests that SapR controls many pathways of unknown function and several known pathways including lipid metabolism and energy utilization.

To understand what genomic regions are activated and repressed by SapR, we analyzed the RNA-seq data set using WoPPER (Puccio et al., 2017). WoPPER is a web tool based on Position RElated genomics Data Analysis (PREDA) with the integration of gene expression data and the physical position of genes along the genome to identify differentially expressed chromosomal regions in bacteria (Puccio et al., 2017). WoPPER analysis identified 72 gene clusters up-regulated and 73 gene clusters down-regulated in the *sapR* mutant strain (Supplementary Figure S5). This analysis highlights the broad reach that SapR has on gene expression throughout the entire genome and indicates that SapR has the ability to activate and repress transcription. This analysis also identifies two nitrogen metabolism gene clusters that are differently regulated by SapR (Supplementary Figure S5; Table 2; Supplementary Table S2). One of these gene clusters (*BP1026B_I1015*–*BP1026B_I1020*) is located on chromosome I (Cluster ID: 21) and up-regulated, while the second (*BP1026B_II1220*, *BP1026B_II1222*-*BP1026B_II1225*) is located on chromosome II (Cluster ID: 30) and down-regulated in the *sapR* mutant. These nitrate reduction gene clusters share 69–79% sequence homology (Mangalea et al., 2017) but have different expression patterns controlled by SapR indicating possible distinct roles for each. This evidence suggests that *Bp* may exploit different nitrogen metabolism operons to survive in diverse environments.

## Discussion

Once being exposed to various environments, the bacterial cell surface is the front line for sensing environmental cues followed by transducing signals inside the cell to alter gene expression and adapt to new conditions. Bacteria have evolved sophisticated regulatory strategies to modulate their cell surface components. Many membrane-associated *Bp* virulence factors, including T6SS-5 and T3SS3, are controlled by transcriptional regulators (Sun et al., 2010; Chen et al., 2011; Lennings et al., 2018). Sap1 was recently identified to be a critical *Bp* attachment factor for intracellular infection (Heacock-Kang et al., 2021), but there is no information on how it is regulated. To identify possible regulators of the *sap1* gene, we first mapped the transcriptional start site of *sap1* and defined its promoter region, which was used for the high-throughput random transposon mutagenesis screen. BP1026B_II1046 (SapR) was identified as a potential regulator for *sap1*. SapR models with 100.0% confidence to *V. vulnificus* NanR that responses specifically to ManNAc-6P the catabolic intermediate of sialic acid present on the mucus layer of human intestines (Roy et al., 2010; Hwang et al., 2013; Kelley et al., 2015). Through the present manuscript we show that SapR has a role in pathogenesis consistent with how NanR regulation is critical for *V. vulnificus* pathogenesis (Hwang et al., 2013). We identified a functional correlation between Sap1 and SapR, where SapR is also important for attachment and infection in RAW264.7 murine macrophage cells. The Δ*sapR* mutant showed impaired attachment efficiency comparable to Δ*sap1* mutant. Its attachment defect was restored and increased to 140% of wildtype when SapR was constitutively expressed on a plasmid. The involvement of SapR during infection was also confirmed by the fact that Δ*sapR* mutant had a significant defect during intracellular replication in macrophage cells. The expression of *sapR* is up-regulated within the endocytic vesicle during intracellular infection, the same intracellular niche that *sap1* is expressed (Heacock-Kang et al., 2021; Supplementary Figure S4). This indicates that *sap1* and *sapR* expression are highly coordinated during *Bp* intracellular infection.

We utilized an RNA-seq approach and identified that SapR regulates genes locally and globally within the *Bp* genome. In addition to showing that SapR regulates genes on both chromosomes, 23% of the SapR regulated proteins are localized to the cellular envelope. *BP1026B_II1045*, the neighboring gene to *sapR*, was highly down-regulated in Δ*sapR* mutant and encodes D-alanyl-D-alanine dipeptidase sharing 33% amino acid identity with *Enterococcus faecium* VanX responsible for vancomycin resistance (Lessard and Walsh, 1999). Vancomycin is an important drug for the treatment of Gram-positive bacterial infections, but is rarely used to treat Gram-negative bacterial infections due to the limited permeability of the outer membrane (Lessard and Walsh, 1999). However, *vanX* homologs in Gram-negative bacteria have been reported in multiple physiological roles including peptidoglycan metabolism that may contribute to preventing activation of the host immune system (Hilbert et al., 1999). SapR also regulates other genes involved in a putative dipeptide transport system for cell survival under starvation conditions (Lessard and Walsh, 1999) including a periplasmic solute-binding protein (BP1026B_II1044), an ABC transporter permease (BP1026B_II1043), a dipeptide transport system permease (BP1026B_II1042), and two ABC transporter ATP-binding proteins (BP1026B_II1041, BP1026B_II1040). Modifying peptidoglycan under stress to reduce intracellular defense stimulation has been exploited by intracellular pathogens to promote adaption to eukaryotic intracellular niche (Chaput et al., 2006; Rico-Pérez et al., 2016; García-Del Portillo, 2020). These data suggest that SapR up-regulates this gene cluster within the endocytic vesicle and it could be a critical step for *Bp* adaptation to intracellular survival. SapR also regulates other membrane-associated genes encoding outer membrane porins (BP1026B_II0620, BP1026B_II0774, BP1026B_II1369, BP1026B_I0046 and BP1026B_I3218), the autotransporter BP1026B_I1153 (BatA), anaerobically induced outer membrane protein BP1026B_II1580 and two TssM proteins BP1026B_II0111 (*tssM-2*) and BP1026B_II2265 (*tssM-6*). BatA localized on the bacterial surface is protective in vaccination against lethal aerosol infection with *Bp* and *Burkholderia mallei* (Lafontaine et al., 2019). TssM protein is a three-transmembrane spanning segment inner membrane protein, directly interacting with TssL by its N-terminal domain (Ma et al., 2009; Zoued et al., 2014). TssM is an essential protein for the function of a sub-complex of the T6SS comprised of four cell envelope proteins TssL, TssM, TagL, and TssJ (Aschtgen et al., 2010; Burtnick et al., 2011; Lennings et al., 2018). Although tssM-2 and tssM-6 belong to T6SS systems that are not generally believed to be important for complete pathogenesis in infecting mammalian cells, our data showing these components are regulated by SapR could provide genomic clues for the identification of its additional functions. In addition, SapR regulates *BP1026B_II1369*, which encodes an outer membrane channel forming protein TolC. This channel is involved in the expulsion of diverse molecules from the cell including protein toxins and antibacterial drugs (Koronakis et al., 2004). TolC is ubiquitous among Gram-negative bacteria, and has been tied to bacterial virulence and pathogenesis in *Enterobacter*, *Borrelia*, *Salmonella*, *Vibrio*, *Legionella*, *Francisella* (Masi and Pagès, 2013). Taken together these data suggest that SapR regulates virulence factors associated with the cell membrane and plays a role in dynamic modification of *Bp* membrane components for adaptation to changes of its residing environment.

Through WoPPER analysis, the *narGHJI-1*/*narK-1* complex (*BP1026B_I1015* - *BP1026B_I1020*; ChI_Cluster ID 21) was shown to be up-regulated in the *sapR* mutant that encodes a nitrate reductase system, responsible for nitrate/nitrite transport and nitrate reduction in the absence of oxygen (Moreno-Vivián et al., 1999; Mangalea et al., 2017; Supplementary Figure S5). The *narX*/*narL* two-component regulatory system upstream of the *narGHJI* operon used in sensing and regulating nitrate metabolism in response to exogenous nitrate (Mangalea et al., 2017) was not influenced by SapR. *BP1026B_II1220* and *BP1026B_II1222*-*BP1026B_II1225* encode another nitrate/nitrite transporter (*narK*) and nitrate reductase complex (*narGHJI-2*) on the second chromosome (ChII_Cluster ID 30; Supplementary Figure S5), respectively. Interestingly, *narGHJI-*2 and *BP1026B_II1220* were significantly down-regulated in the *sapR* mutant suggesting that this gene cluster is activated by SapR. This evidence indicates that *Bp* may exploit different nitrogen metabolism operons to survive in diverse environments.

In summary, this study has identified SapR as a positive transcriptional regulator for Sap1 and shown that SapR regulates membrane-associated virulence factors, peptidoglycan, lipid, and nitrogen metabolism. Future investigation on the genome wide binding sites of SapR will aid in a better understanding of the molecular mechanism of SapR regulation. Overall, our data presented here contribute to valuable insights into the complex regulation network during *Bp* intracellular infection.

## Data availability statement

The data presented in the study are deposited in the GEO repository, accession number GSE220579.

## Author contributions

ZS, YH-K, and TH designed the experiments. ZS, YH-K, DC, and JZ-S conducted the experiments. ZS, YH-K, IM, and TH analyzed the data and wrote the manuscript. All authors contributed to the article and approved the submitted version.

## Funding

This project was supported by the United States National Institutes of Health (NIH)/National Institute of Allergy and Infectious Diseases (NIAID) grant number R21AI123913 awarded to TH.

## Conflict of interest

The authors declare that the research was conducted in the absence of any commercial or financial relationships that could be construed as a potential conflict of interest.

## Publisher’s note

All claims expressed in this article are solely those of the authors and do not necessarily represent those of their affiliated organizations, or those of the publisher, the editors and the reviewers. Any product that may be evaluated in this article, or claim that may be made by its manufacturer, is not guaranteed or endorsed by the publisher.

## Footnotes

^1^ http://www.fruitfly.org/seq_tools/promoter.html

^2^ http://www.idtdna.com
