# Supplementary figures and images for: A virulence activator of a surface attachment protein in Burkholderia pseudomallei acts as a global regulator of other membrane-associated virulence factors
Source: Front Microbiol. 2023 Jan 16;13:1063287. doi: 10.3389/fmicb.2022.1063287 (PMC9884982; doi:10.3389/fmicb.2022.1063287)

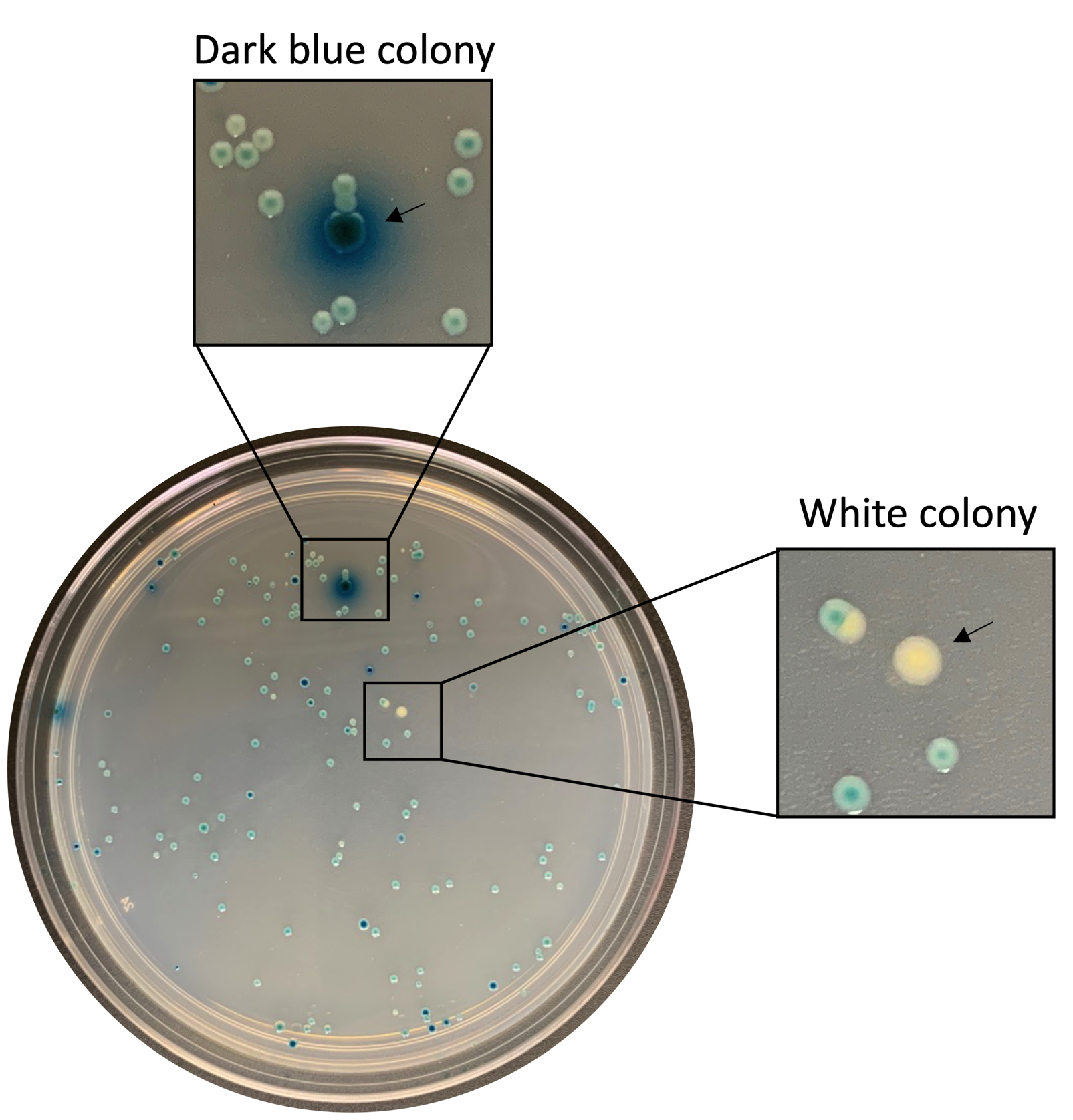

Supplement: Supplementary file 1 [file Image_1.TIFF]

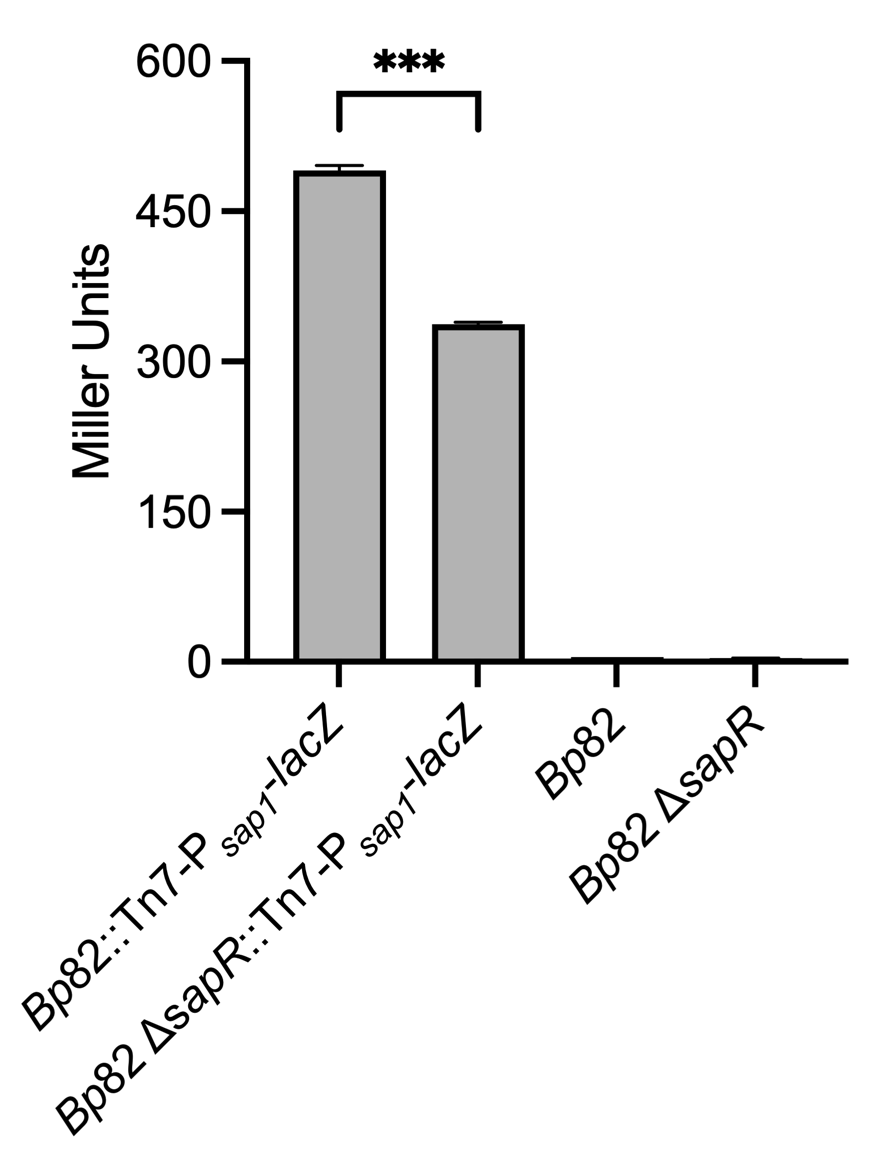

Supplement: Supplementary file 2 [file Image_2.TIFF]

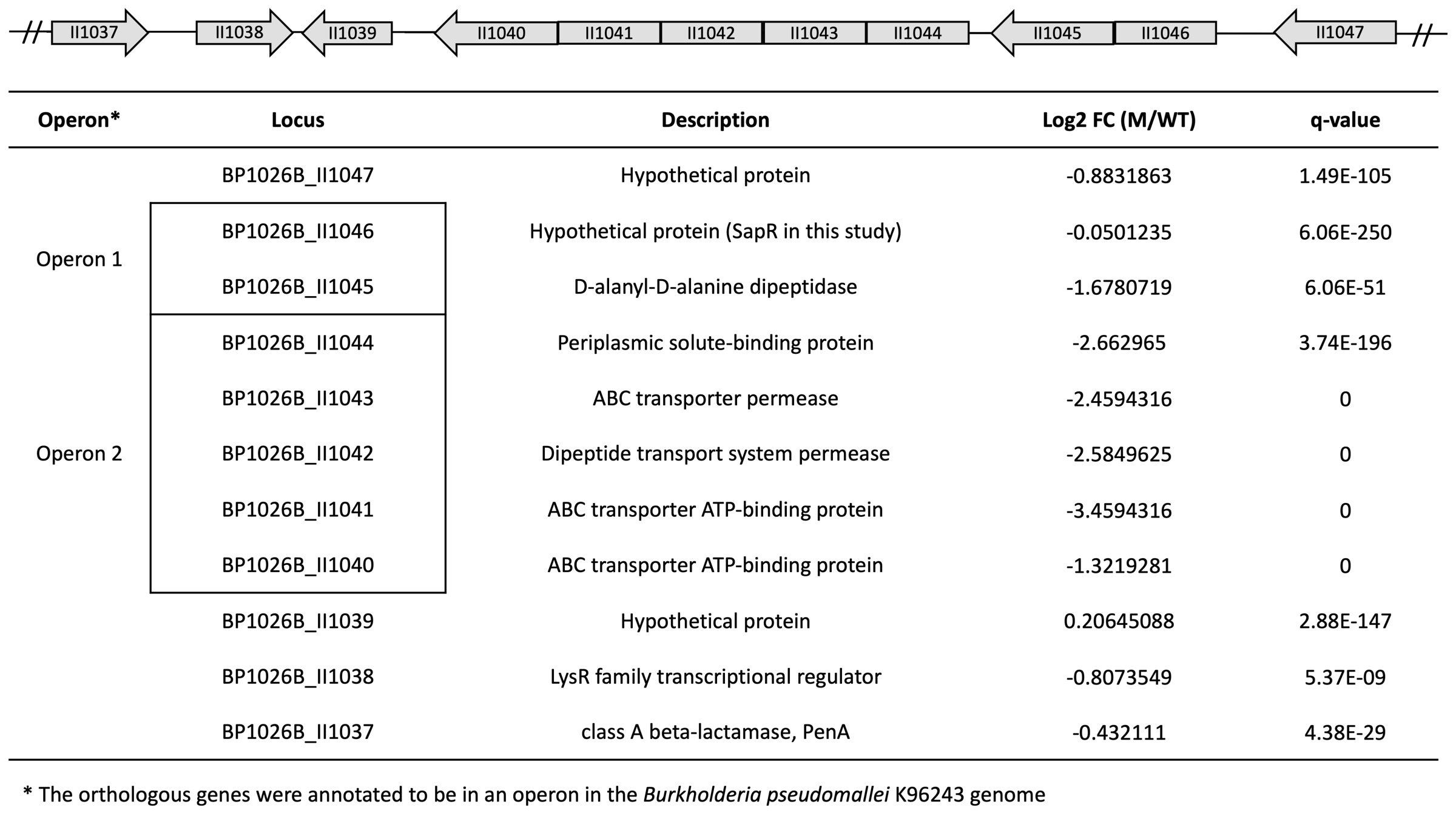

Supplement: Supplementary file 3 [file Image_3.TIFF]

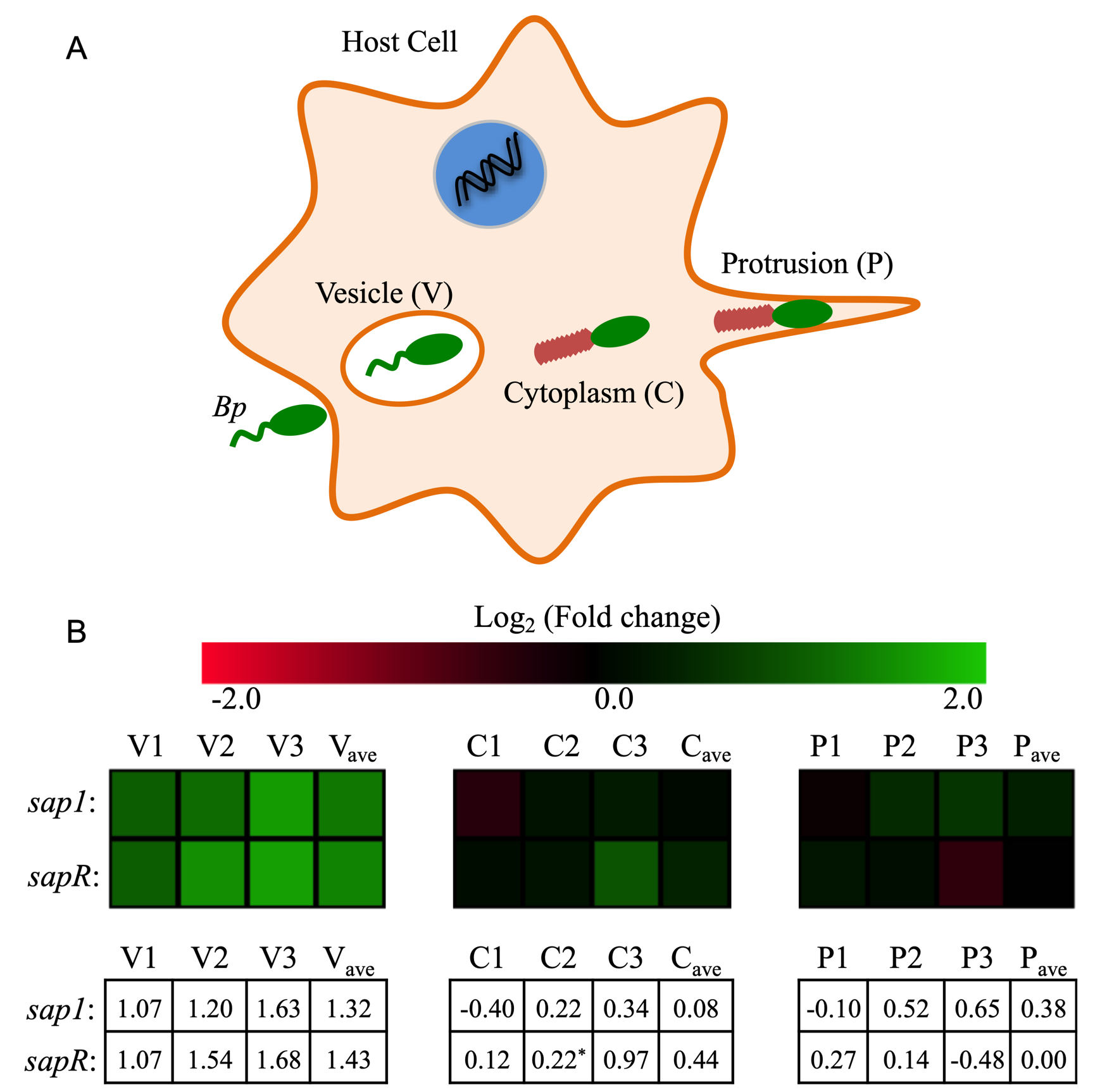

Supplement: Supplementary file 4 [file Image_4.TIFF]

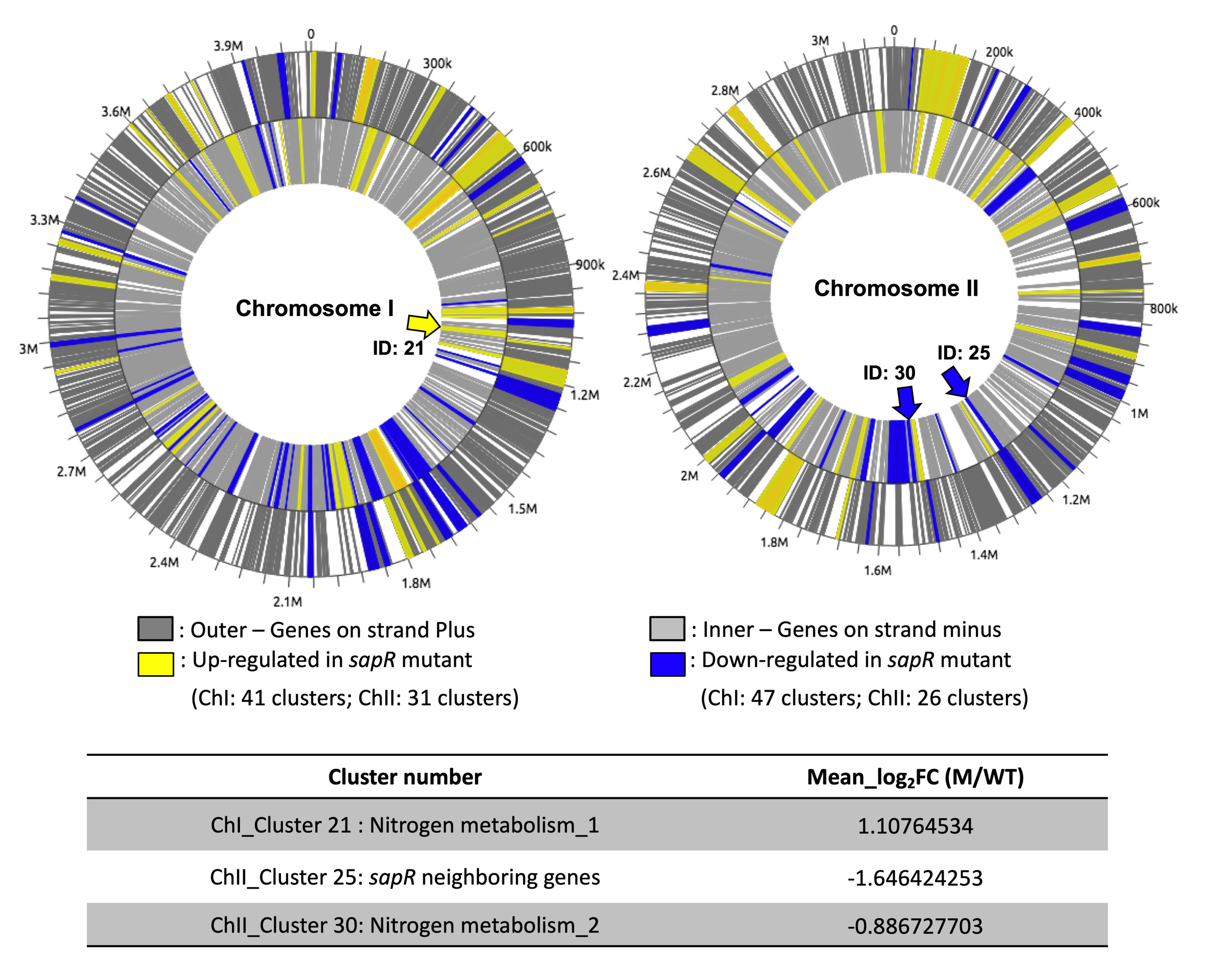

Supplement: Supplementary file 5 [file Image_5.TIFF]
